# Clinical characteristics and outcomes for patients with non‑metastatic castration-resistant prostate cancer

**DOI:** 10.1038/s41598-021-01042-7

**Published:** 2021-11-12

**Authors:** Peter Arnold, Maria Cristina Penaloza-Ramos, Lola Adedokun, Sarah Rees, Mohamed Lockhat, Lisa Spary, Alan Watkins, Vincent Gnanapragasam, Simon J. Crabb

**Affiliations:** 1 SAIL Databank, Swansea University Medical School, Swansea, UK; 2 Janssen-Cilag Ltd, 50-100 Holmers Farm Way, High Wycombe, Buckinghamshire, HP12 4EG UK; 3 Divison of Urology, Department of Surgery & Cambridge Urology Translational Research and Clinical Trials Office, University of Cambridge, Cambridge, UK; 4 Cancer Sciences Unit, University of Southampton, Southampton, UK

**Keywords:** Cancer epidemiology, Urological cancer, Prostate cancer

## Abstract

This study used linked, routinely-collected datasets to explore incidence, clinical characteristics and outcomes of prostate cancer (PC) patients who experience a rise in prostate-specific antigen (PSA) levels despite androgen deprivation therapy (ADT), without evidence of metastases in their patient record, termed non-metastatic castration-resistant PC (nmCRPC). Routinely collected administrative data in Wales were used to identify patients diagnosed with PC and nmCRPC from 2000–2015. Logrank tests and Cox proportional hazard models were used to compare time-to-events across subgroups defined by PSA doubling time and age. Of 38,021 patients identified with PC, 1,465 met nmCRPC criteria. PC incidence increased over the study period, while nmCRPC categorizations reduced. Median time from PC diagnosis to nmCRPC categorization was 3.07 years (95% confidence interval [CI] 2.91–3.26) and from nmCRPC categorization to metastases/death was 2.86 years (95% CI 2.67–3.09). Shorter PSA doubling time (≤ 10 months, versus > 10 months) was associated with reduced time to metastases or death (2.11 years [95% CI 1.92–2.30] versus 5.22 years [95% CI 4.87–5.51]). Age was not significantly associated with time to metastases/death. Our findings highlight key clinical characteristics and outcomes for patients with nmCRPC prior to the introduction of recently approved treatments.

## Introduction

Prostate cancer (PC) is the second most frequent malignancy in men worldwide, with over 48,000 incident cases in the UK in 2017, 2,705 of which occurred in Wales^1,2^. Palliative treatment options for patients with PC are based around androgen deprivation therapy (ADT), which initially elicits a response in most patients with castration-sensitive prostate cancer (CSPC)^3^. However, despite an androgen-deprived state, a subset of men treated with ADT subsequently experience rising prostate-specific antigen (PSA) levels, indicating a biochemical recurrence or progression of disease termed castration-resistant prostate cancer (CRPC). Typically, this occurs with co-existent radiographic evidence of progressive distant metastatic disease (mCRPC). However, transition to CRPC may also occur without radiologically detectable distant metastases, classified as non-metastatic CRPC (nmCRPC)^4^.

In combination with ADT, established treatment options for the clinical management of CSPC or mCRPC include docetaxel chemotherapy, the androgen synthesis inhibitor therapy abiraterone acetate plus prednisone, and the novel androgen receptor antagonists enzalutamide and apalutamide^5–7^. Recent data also support the use of enzalutamide, apalutamide, and darolutamide in patients with nmCRPC and a PSA doubling time of ≤ 10 months, on the basis of a metastasis-free survival benefit^8–10^. These relatively recent therapeutic options for nmCRPC highlight the importance of documenting accurate real-world incidence, clinical characteristics and outcomes to inform treatment needs.

There are, however, very limited data available on the burden of nmCRPC disease outside randomized controlled trials (RCTs; SPARTAN, PROSPER and ARAMIS)^8–10^. While RCTs are critical to demonstrating the safety and efficacy of new treatments, their design and inclusion criteria, including increased imaging requirements and relatively higher risk patients based on PSA doubling time, can affect documented patient outcomes and limit generalization to real-world populations. Real-world evidence, using data collected from routine clinical settings and registries, can complement RCTs and support their generalization to broader patient groups. Previous publications exploring nmCRPC and mCRPC in real-world settings have mostly included small samples of patients from specific hospitals or data registries^11,12^. Recently, a population-based observational study in Sweden reported clinical characteristics and outcomes of over 1,700 patients with nmCRPC over 10 years^13^. To address this paucity of data, we undertook a retrospective analysis of an nmCRPC patient cohort using Welsh health database records between 2000–2015. We aimed to describe key clinical characteristics and outcomes of this patient population and identify potential risk factors for early disease progression or mortality.

## Materials (patients) and methods

### Study design and data sources

This retrospective observational cohort study was conducted using routinely collected administrative data in Wales, including prescriptions, diagnostic procedures and hospital admissions, hosted by the Secure Anonymised Information Linkage (SAIL) Databank at Swansea University^14,15^. The study population denominator was the Welsh Demographics Service (WDS) dataset, containing data for all individuals registered with a Welsh general practitioner (GP), including registration start and end dates^16^. Clinical information arising from National Health Service (NHS)-funded services (which are free to patients at the point of delivery) provided to Welsh residents was derived from Wales Primary Care GPs (WLGP), Welsh Cancer Incidence and Surveillance Unit (WCISU) and Patient Episode Dataset for Wales (PEDW). Date and cause of death was derived from the Office for National Statistics (ONS) Annual District Deaths Extract (ADDE), which includes details of all deaths of Welsh-domiciled residents. Apart from the WLGP dataset, coverage for each dataset includes all of Wales, estimated to include approximately 1.5 million males in 2019^17^.

The PEDW and WCISU datasets were used to identify patients with PC using International Classification of Diseases (ICD)-10 codes, while the WLGP dataset used Read version 2 codes. Each of these systems act as a coded thesaurus of clinical terms to provide standard vocabulary for clinicians; the ICD system is globally recognized^18^ while the Read system is used primarily within the NHS^19^. An algorithm, implemented at two levels of stringency, was designed to identify nmCRPC cases based on the presence (in the GP record) of codes for receipt of ADT (luteinizing hormone-releasing hormone [LHRH] analogue, LHRH antagonist or bilateral surgical castration), PSA test results and codes indicating presence or absence of metastatic disease. Full details on dataset use are available in the supplementary material.

### Patients

This analysis features three nested patient cohorts identified from the linked datasets: a PC cohort, the ‘main nmCRPC cohort’, and a ‘stringent nmCRPC cohort’, based on supplementary inclusion criteria to those used for the main nmCRPC cohort. The stringent nmCRPC subset was used to validate results from the main nmCRPC cohort, given that explicit non-metastatic PEDW codes were not available for many patients and most diagnoses were inferred from available medical records.

The PC cohort had a diagnosis of PC made between 01 January 2000 and 31 December 2015. Follow-up data were available until 31 March 2018. The main nmCRPC cohort included all patients from the PC cohort with ≥ 1 period of ADT in their GP history after PC diagnosis and at least three subsequently increasing PSA measurements that met the Prostate Cancer Working Group 2 criteria for PSA progression^20^. These criteria required two rises in PSA values with ≥ 1 week between readings and a final reading ≥ 2 ng/ml; these measurements were also used to determine PSA doubling time^20^. The nmCRPC categorization date was taken to be the date of the first rising PSA test (i.e. the second test overall). Patients with any other primary or secondary malignant cancer, receipt of a treatment for metastatic disease in their GP history (secondary care prescribing data were not available), or presence of a metastatic disease morphology code before their second qualifying PSA measurement, were excluded from the nmCRPC cohort.

The stringent nmCRPC subset also required an available morphology code explicitly confirming non-metastatic disease status (in the absence of any codes indicating metastatic disease) up to the point of meeting criteria for nmCRPC (from PEDW, up to date of inclusion).

### Statistical analysis

Analyses of survival and Kaplan–Meier plotting were performed using R (V4.0.2). Logrank tests and Cox proportional hazard models were used to compare time-to-event analyses from diagnosis of PC to nmCRPC categorization and from nmCRPC categorization to metastasis or death according to PSA doubling time (≤ 10 months, > 10 months) and age groups (≤ 70, > 70– ≤ 75, > 75– ≤ 80, > 80 years). Continuous variables are described using means and standard deviation; categorical variables are summarized with frequency counts and proportions.

### Ethical approval

This study was approved by the SAIL Information Governance Review Panel (IGRP) under project number 0775. Approval for the use of anonymized data in this study (SAIL project number 0775), provisioned within the SAIL Databank, was granted by an independent IGRP with membership comprising senior representatives from the British Medical Association (BMA), the National Research Ethics Service (NRES), Public Health Wales and NHS Wales Informatics Service (NWIS). All methods were carried out in accordance with the relevant guidelines and regulations.

Written informed consent was not required for this study, as it used anonymized, routinely collected data. The use of anonymized data for research is outside the scope of the EU General Data Protection Regulations (GDPR) and the UK Data Protection Act.

## Results

### Patient demographics and baseline characteristics

Patient demographics and baseline characteristics are shown in Table 1. Between 01 January 2000 and 31 December 2015, 38,021 men were diagnosed with PC, of which 1,465 (3.9%) were further categorized with nmCRPC based on our main cohort definition, and 470 (1.2%) patients were categorized with nmCRPC using the stringent subset definition (Supplementary Figure S1). Compared to the overall PC cohort, the median age of the main nmCRPC cohort was approximately five years greater at initial PC diagnosis (70.6 vs 75.6 years), at commencement of ADT (71.8 vs 76.6 years), at the point of development of metastatic disease (71.9 vs 78.1 years) and at death (76.9 vs 83.2 years; Table 1).

**Table 1:** Patient demographics and baseline characteristics.

	All patients with PC (N = 38,021)	Main nmCRPC cohort (n = 1,465)	All patients with PC excluding main nmCRPC cohort (n = 36,556)
**Age at, median (IQR)**			
PC diagnosis	70.6 (65.0–76.9)	75.6 (69.2–80.4)	72.7 (65.9–79.6)
Initiation of ADT	71.8 (66.3–77.6)	76.6 (70.9–81.3)	74.3 (68.1–79.2)
nmCRPC diagnosis	N/A	79.5 (73.6–84.6)	N/A
Development of metastases	71.9 (65.9–77.5)	78.1 (72.3–84.3)	75.4 (68.8–81.5)
Death	76.9 (70.8–83.0)	83.2 (77.0–88.4)	82.0 (75.8–87.1)
**Treatment, n (%)**			
ADT	16,911 (44.5)	1,465 (100.0)	15,446 (42.3)
Orchidectomy	220 (0.6)	< 5 (< 0.1)^a^	> 215 (~ 0.6)
Prostatectomy	4,361 (11.5)	18 (1.2)	4,343 (11.9)
Radiotherapy	5,341 (14.0)	189 (12.9)	5,152 (14.1)
PSA doubling time			
≤ 10 months	N/A	952 (65.0)	N/A
> 10 months	N/A	513 (35.0)	N/A
**Gleason score**			
≤ 6	9,407 (24.7)	187 (12.8)	9,220 (25.2)
7	6,726 (17.7)	269 (18.4)	6,457 (17.7)
8–10	4,570 (12.0)	347 (23.7)	4,223 (11.6)
Unknown	17,318 (45.6)	662 (45.2)	16,656 (45.3)
**TNM score**			
Tany N0 M0	5,600 (14.7)	64 (4.4)	5,536 (15.1)
Tany N1–2 M0	269 (0.7)	N/A	269 (0.7)
Tany Nany M1	619 (1.6)	N/A	619 (1.7)
Other	84 (0.2)	N/A	84 (0.2)
Unknown	31,449 (82.7)	1,401 (95.6)	30,048 (82.2)
**Time in months, median (IQR)**			
From PC diagnosis to nmCRPC	N/A	37.4 (20.3–66.9)	N/A
Duration of follow-up from nmCRPC diagnosis	N/A	37.6 (19.5–61.3)	N/A

ADT: androgen deprivation therapy; IQR: interquartile range; N/A: not available; nmCRPC: non-metastatic castration-resistant prostate cancer; PC: prostate cancer; PSA: prostate-specific antigen; TNM: tumor, node, metastasis.

^a^This result is presented as ‘ < 5’ to ensure patient anonymity.

Patient demographics and baseline characteristics for the stringent nmCRPC cohort are available in Supplementary Table S1.

### Incidence of PC and nmCRPC

**Figure 1:**
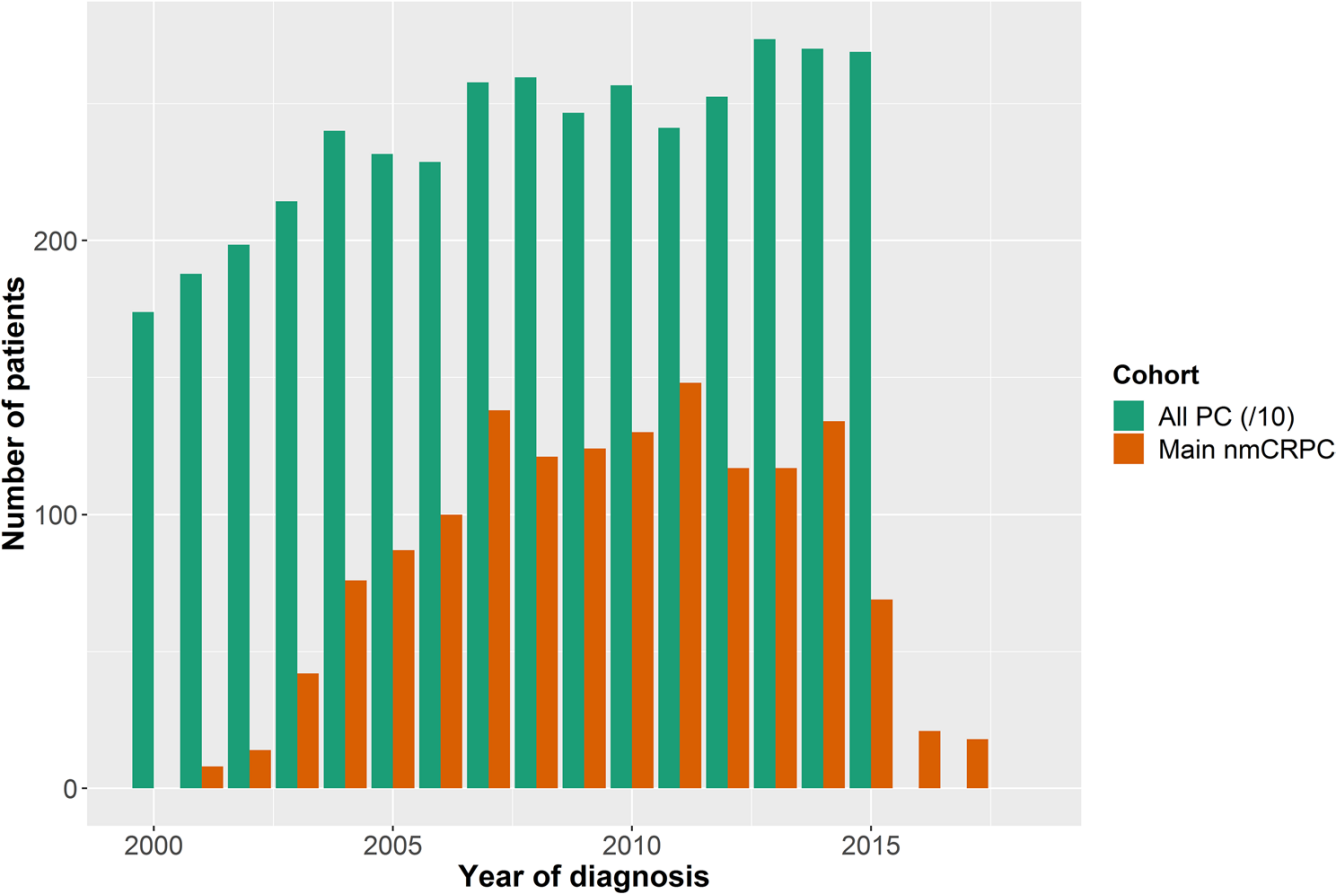
Incidence of PC and nmCRPC (for individuals diagnosed with PC from 2000–2015). Incidence of PC is defined as the number of patients with a first PC diagnosis in each year. Incidence of nmCRPC is the number of patients with a PC diagnosis who have received ADT and who have ≥ 3 PSA tests which indicate nmCRPC. The date of nmCRPC incidence is the first test showing an increase in PSA, i.e. the second test overall. The third PSA test confirms the increase. Incidence of PC has been divided by 10 to allow for easy comparison with the incidence of nmCRPC. ADT: androgen depravation therapy; nmCRPC: non-metastatic castration-resistant prostate cancer; PC: prostate cancer; PSA: prostate-specific antigen.

**Figure 2:**
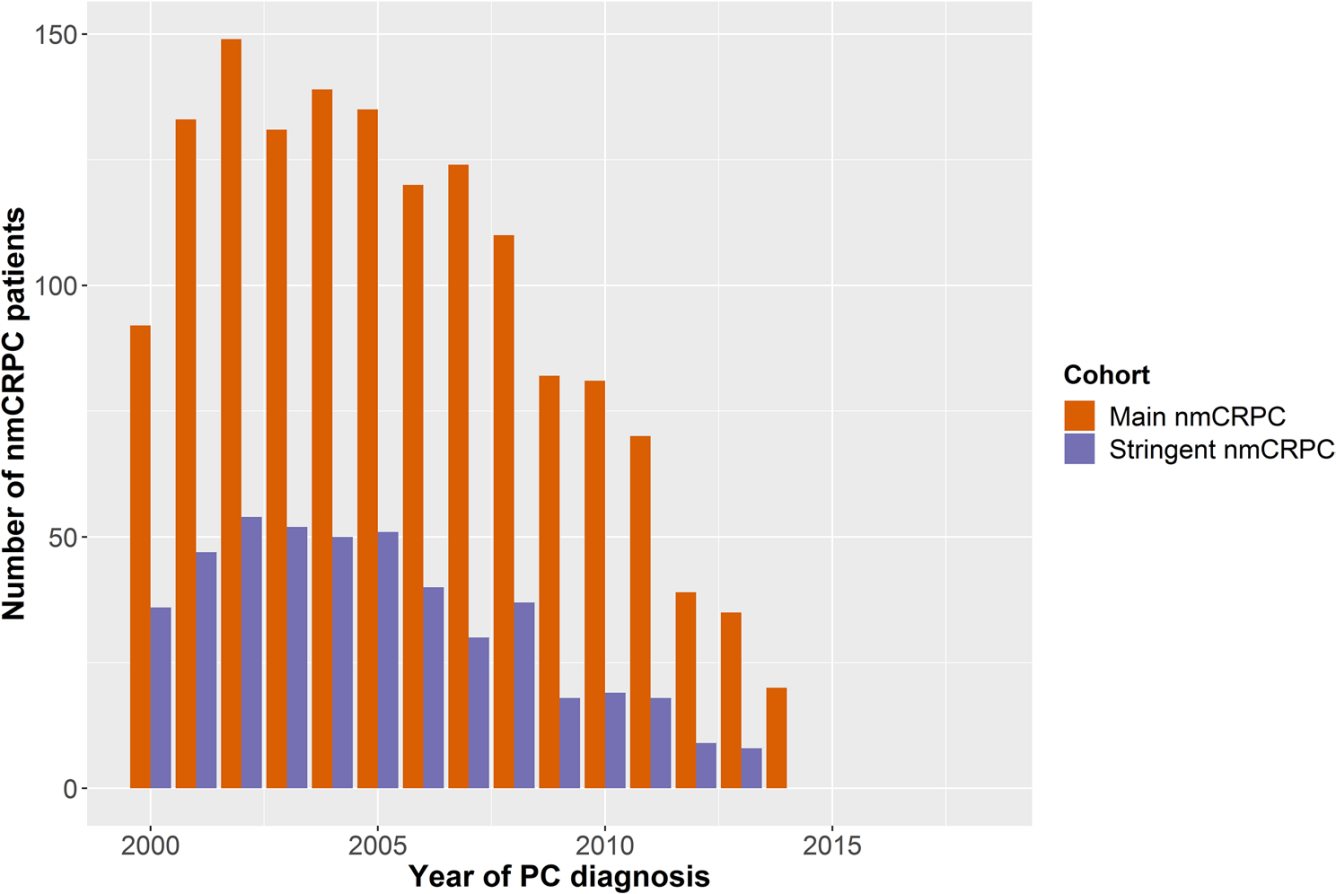
Number of patients diagnosed with PC each year who later progressed to meet inclusion criteria for the main and stringent nmCRPC cohorts. The patients in each column of this graph had a PC diagnosis in the year specified on the x-axis. nmCRPC: non-metastatic castration-resistant prostate cancer; PC: prostate cancer.

PC incidence in Wales, defined as the number of patients with a first PC diagnosis, increased from 1,739 in 2000 to 2,689 in 2015 (Fig. 1). The nmCRPC incidence in the main nmCRPC cohort (defined as the number of patients with a PC diagnosis who have received ADT and who have at least three PSA tests which indicate nmCRPC, as per the requirements outlined previously) increased from 2000–2007 and then remained relatively stable. However, from 2015 onwards the number of new nmCRPC cases reduced until only 18 cases were observed in 2017 (Fig. 1). The numbers of patients diagnosed with PC later progressing to meeting the inclusion criteria for the main and stringent nmCRPC cohorts reduced considerably from 2007 (Fig. 2).

### Overall clinical outcomes

**Figure 3:**
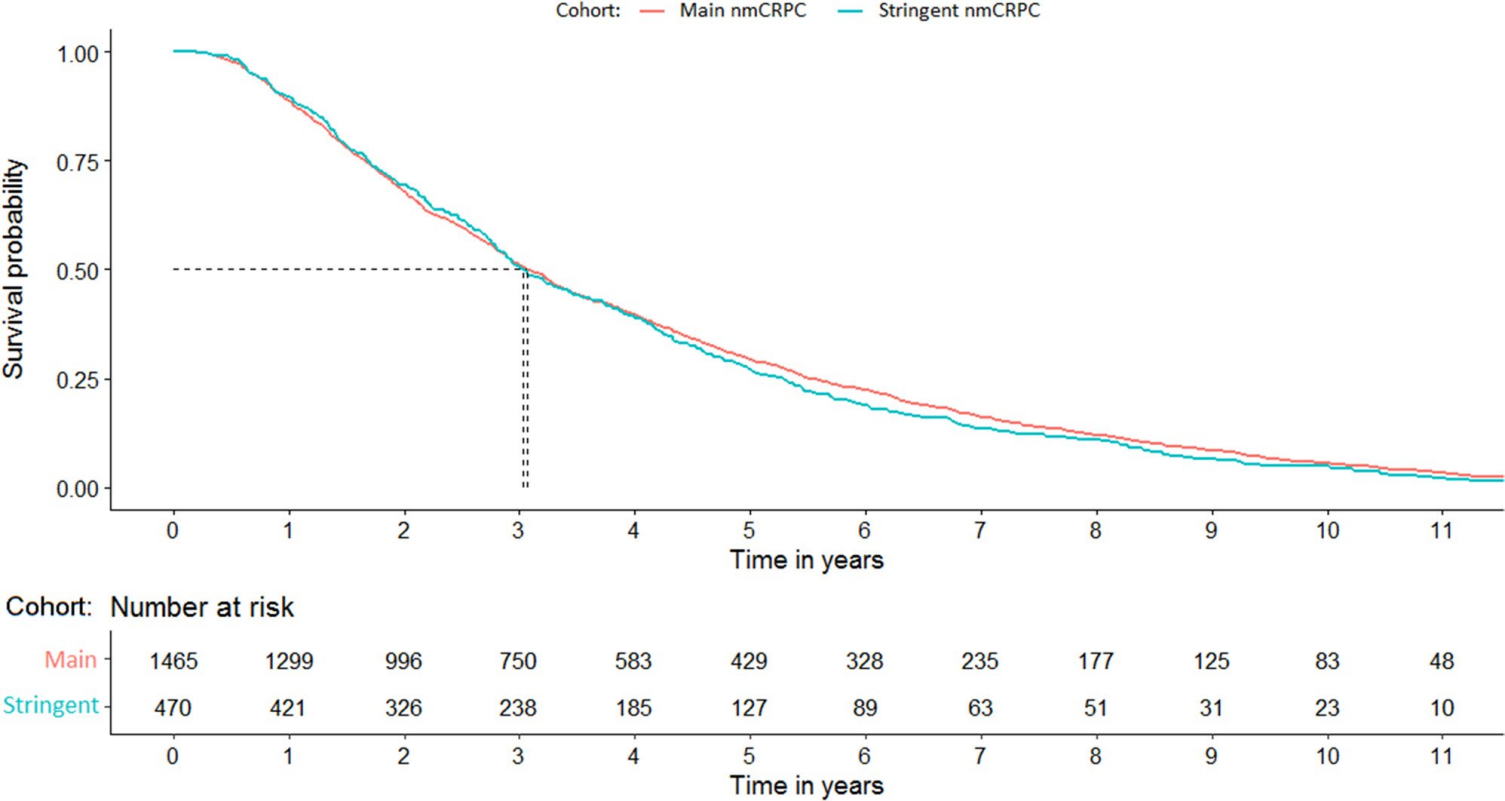
Time from initial PC diagnosis to meeting inclusion criteria for the main and stringent nmCRPC cohorts. nmCRPC: non-metastatic castration-resistant prostate cancer; PC: prostate cancer.

For the main nmCRPC cohort, median time from PC diagnosis to nmCRPC categorization was 3.07 years (95% confidence intervals [CI] 2.91–3.26; Fig. 3) with only 429 (29.3%) patients living with a PC diagnosis for ≥ 5 years before progressing to nmCRPC. The corresponding results for the stringent nmCRPC subset were consistent with the main nmCRPC cohort (Fig. 3).

**Figure 4:**
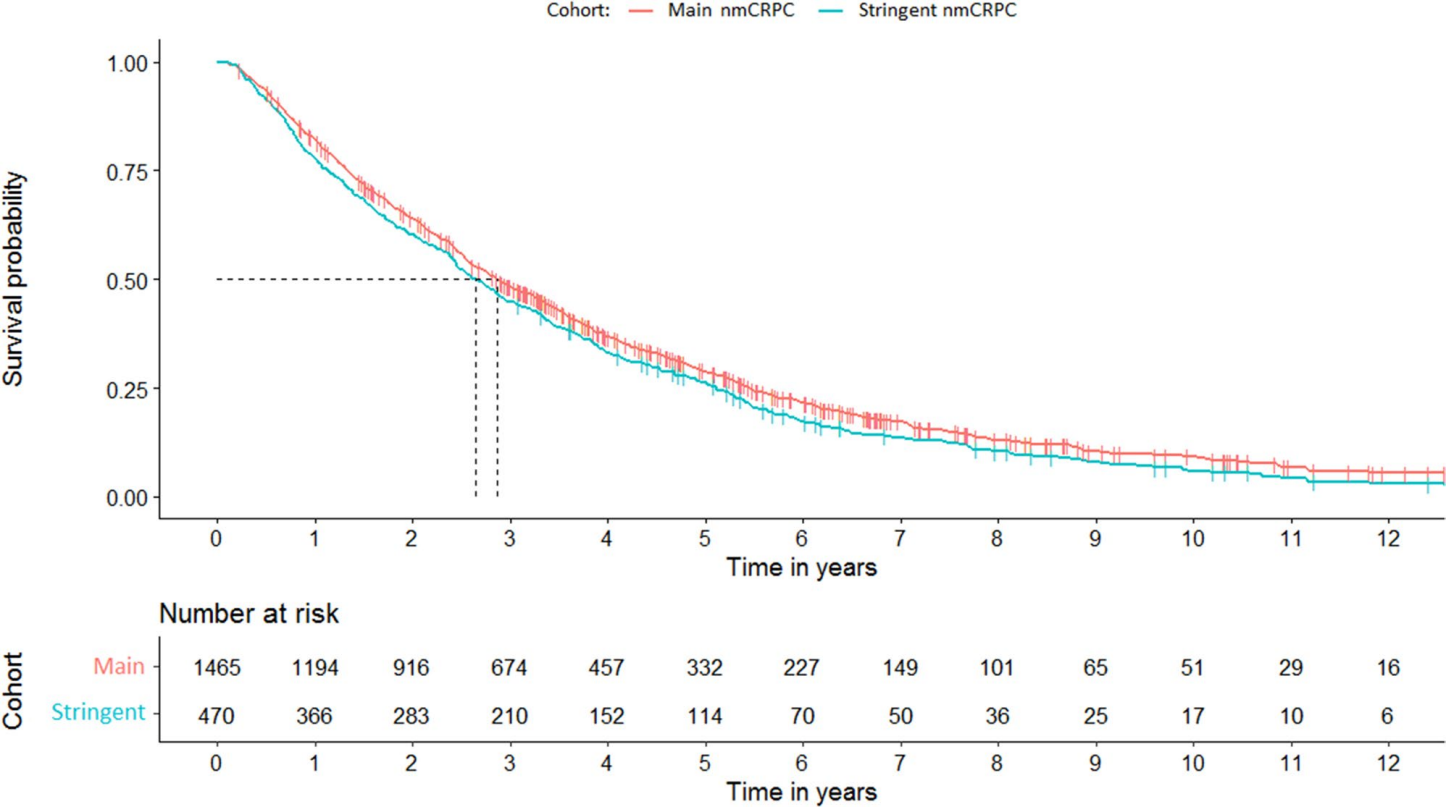
Time from meeting inclusion criteria for the main and stringent nmCRPC cohorts to metastases or death. nmCRPC: non-metastatic castration-resistant prostate cancer.

Patients in the main nmCRPC cohort had a median time from nmCRPC categorization to metastases or death of 2.86 years (95% CI 2.67–3.09) and a 28.9% chance of surviving 5 years without metastases or death. After 5 years, 332 patients in this cohort were still at risk (were not censored and surviving without metastases or death, Fig. 4). Results from the stringent nmCRPC subset were generally comparable to those of the main nmCRPC cohort, but with a lower median survival time of 2.65 years (95% CI 2.41–2.95).

### Clinical outcomes by PSA doubling time and age group

**Figure 5:**
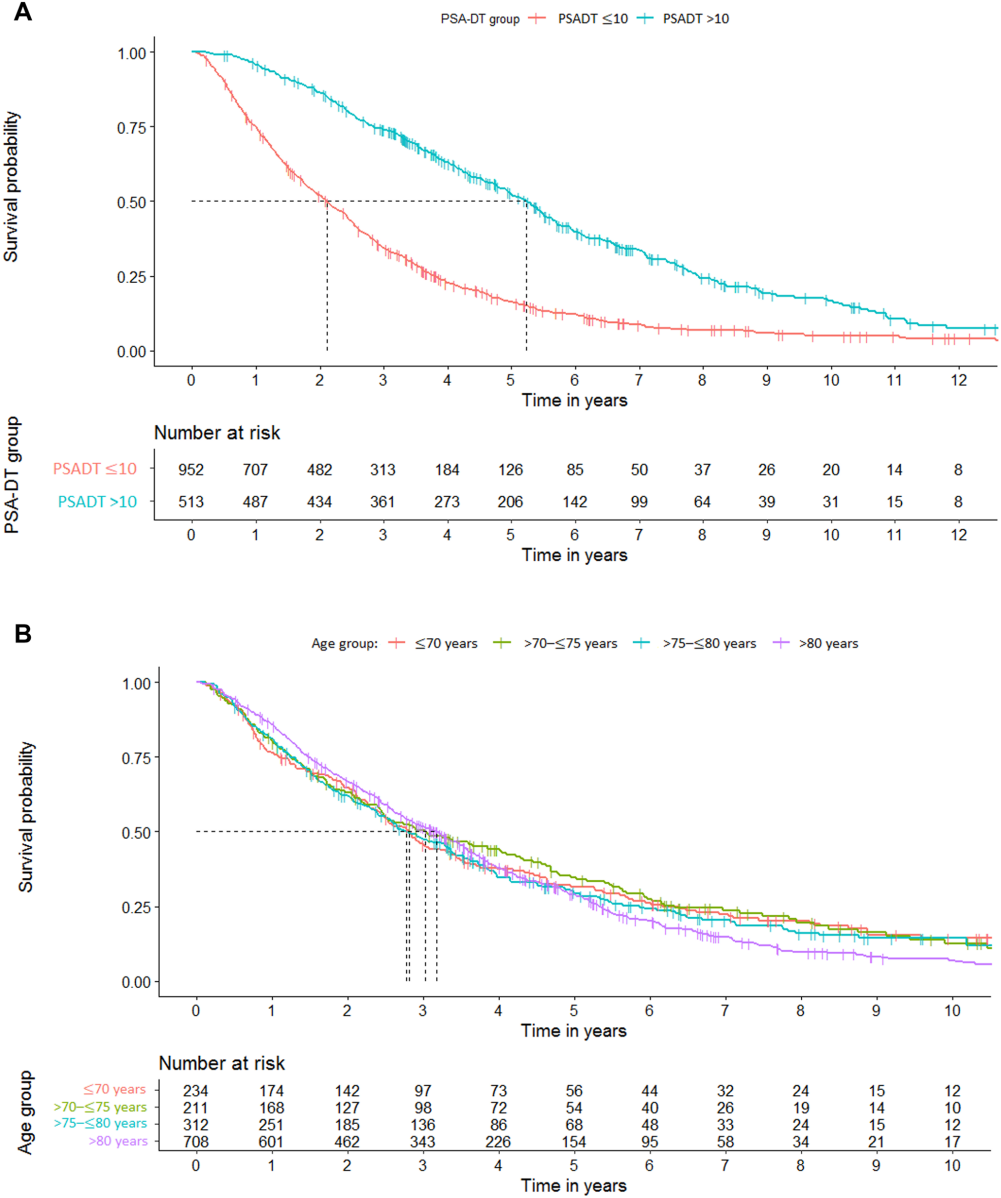
Time to metastases or death, stratified by a) risk group and b) age group. Risk was categorized into two groups, defined as those with a PDADT of either ≤ 10 months or > 10 months. PSA: prostate-specific antigen; PSADT: prostate-specific antigen doubling time.

The effect of PSA doubling time on survival to metastases or death in the main nmCRPC cohort was investigated by stratifying patients by PSA doubling time (≤ 10 months, > 10 months; Fig. 5a). The median time of survival to metastases or death in the ≤ 10 months group was over 3 years shorter than that of the > 10 months group (2.11 years [95% CI 1.92–2.30] vs 5.22 years [95% CI 4.87–5.51]). The percentage of patients reaching 5 years without metastases or death was also lower in the ≤ 10 months group (16.3% [n = 126] vs 52.4% [n = 206]). A Cox regression model was applied to both survival datasets, but testing of the Schoenfeld residuals concluded that the proportional hazard assumption may not hold, therefore results should be treated with caution.

The effect of age on survival to metastases or death was also assessed using a time-to-event analysis (Fig. 5b). Using the Logrank test and the Cox regression model, no significant differences were found between age groups.

## Discussion

This retrospective analysis used linked healthcare datasets to identify and characterize a cohort of patients with nmCRPC in Wales, building upon a limited pool of real-world literature.

The incidence of PC diagnoses rose throughout the study period. Similarly, the incidence of nmCRPC cases appeared to increase from 2000, as expected with the natural progression of patients from PC diagnosis to nmCRPC. However, from 2015 there was a sharp decrease in nmCRPC incidence, which may have been influenced by increased access to, and utilization of, cross-sectional imaging and bone scintigraphy throughout the time period studied^21^. When comparing by year of PC diagnosis, the number of patients with PC which went on to be categorized as nmCRPC started to decrease from 2007 onwards. This may be partially attributed to patients diagnosed in later years of the study not yet reaching nmCRPC.

Wider use of novel imaging techniques as a diagnostic tool in nmCRPC may lead to differing findings in future studies of a similar nature. More accurate detection of metastases can be made using novel imaging^22,23^, which in turn should lead to more accurate diagnoses of nmCRPC and so we can hope to see treatments better tailored to patients’ individual needs in future. As such, we may continue to see decreased nmCRPC incidence, but better clinical outcomes in those who do receive a diagnosis. Furthermore, increased availability of treatments suitable for nmCRPC can be expected to further improve patient outcomes; examples include apalutamide, an androgen receptor inhibitor approved by NICE in September 2021, including for the treatment of high-risk prostate cancer which no longer responds to hormone therapy^24^.

Here, the median time from PC diagnosis to nmCRPC inclusion for the main nmCRPC cohort (3.07 years) was comparable to previous reports, which generally fall between 2–3 years^3,25^. The median time from nmCRPC to metastases or death was 2.86 years, with approximately 30% of patients surviving 5 years without metastases or death. This finding is similar to that of a population-based study of Swedish patients with nmCRPC^13^. A higher percentage of men achieving metastasis-free survival for 5 years after nmCRPC was previously reported in the population-based SEARCH study, although this appeared not to include men who died before developing metastases^11^. The study also required available imaging data and had a smaller sample size, which may account for survival differences^11^.

A time to metastases or death of 2.11 years was reported for patients in this study with a PSA doubling time ≤ 10 months, longer than that reported for the equivalent placebo-treated populations in the SPARTAN (1.35 years), PROSPER (1.23 years) and ARAMIS trials (1.53 years)^8–10^. We have compared this study data to placebo data from the clinical trials because the life-extending treatments evaluated in the trials were not approved for clinical use for most of our study period. However, patients from the SPARTAN and ARAMIS trials had a median time from initial diagnosis to randomization of approximately 7 years (unreported in the PROSPER trial), so many patients in the trial population may have had a much longer time to metastases or death than patients in this real-world cohort^9,10^.

Another key difference between the patient populations is access to imaging during treatment. In the RCTs, comprehensive imaging was carried out at baseline (including computerized tomography [CT] and MRI scans), with any patients with detectable metastases excluded^8–10^. Patients continued to receive imaging at regular timepoints during trial participation, likely outside of real-world practice patterns. Our study had no imaging inclusion requirements and imaging was only recorded from 2007 onwards. This may have affected the reported median time to metastases and death of patients, underlining our initial premise of exploring the real-world clinical characteristics and outcomes of nmCRPC.

Although the effect of PSA doubling time on progression to metastases or death was not statistically significant, the numerical evidence suggested a trend that supported previous conclusions that a shorter PSA doubling time is a predictor of faster disease progression^26,27^. Age at PC diagnosis was not shown to have a significant effect on patterns of progression to metastases or death in this study (for those with nmCRPC). Previous literature in this area is limited, although it has been reported that older age (> 80 years vs < 70 years) in patients with nmCRPC may be associated with higher all-cause mortality^13^.

This retrospective analysis demonstrated that linked datasets allow for the identification of a substantial sample size of patients with nmCRPC based on PSA criteria, which has previously been difficult to establish from database records due to a lack of clinical coding^28^. The analysis benefited from a long follow-up period, capturing the complete journey of many patients from PC diagnosis to metastasis or death. However, retrospective studies of population-based data can have data quality and availability limitations. For example, very few patients had tumor, node and metastasis (TNM) staging data available due to limited coverage, which prevented disease staging. We were also unable to confirm metastatic/non-metastatic disease for all patients due to the cessation of morphology code recording in PEDW from 2017. Given that treatment for metastatic disease was not approved for most of our study period, GP prescriptions indicating metastatic treatment were also unavailable for patients prior to 2013 and secondary care prescribing data were unavailable. Despite these limitations, the main nmCRPC cohort was considered to have successfully included patients with nmCRPC, given that results from the stringent nmCRPC subset generally aligned with the main cohort.

More general limitations of registry data include duplication of records, which can arise due to the anonymous nature of the data^29^, or if the same cancer is diagnosed and reported by more than one provider, as is the case if a clinician/patient seeks a second opinion^30^. Other issues can be the lack of control population, the potential for incomplete follow-up and underestimation of the rate of events due to the absence of rigorous monitoring seen in RCTs^29^. Registry data does, however, allow for a more accurate representation of real-world practice, giving a truer reflection of the effectiveness of treatments and outcomes outside of the rigorously monitored environments of RCTs, which do not account for any additional factors influencing clinician judgement and decisions. Furthermore, the large sample size can also provide more generalizable evidence in comparison to RCTs^29^, which tend to have far smaller sample sizes and strict inclusion criteria, which may include specific treatments, or exclude patients with more complex disease or comorbidities.

This study is one of the first to evaluate the presence of nmCRPC in a real-world, UK-based setting. Our findings provide data on clinical demographics and outcomes in nmCRPC prior to the introduction of novel treatments such as apalutamide, darolutamide and enzalutamide and hence the potential scope for therapeutic need. Future work is required to assess changes in the clinical management of nmCRPC and the impact of these new treatment options on survival outcomes.

## Supplementary Information

Supplementary Information.

## Data Availability

The datasets generated for this study are drawn from patient records held by the SAIL Databank and will not be made publicly available. Data may be accessible upon application to the SAIL databank, subject to SAIL governance processes. Details of this are available on the SAIL website (https://saildatabank.com/application-process/).
