# Supplementary material for: Clinical characteristics and outcomes for patients with non‑metastatic castration-resistant prostate cancer
Source: Sci Rep. 2021 Nov 12;11:22151. doi: 10.1038/s41598-021-01042-7 (PMC8589838; doi:10.1038/s41598-021-01042-7)
Supplement: Supplementary file 1 — Supplementary Information. [file 41598_2021_1042_MOESM1_ESM.pdf]

Clinical Characteristics and Outcomes for Patients with Non-Metastatic Castration-Resistant Prostate Cancer

Peter Arnold, PhD,[1] Maria Cristina Penaloza-Ramos, PhD,[2*] Lola Adedokun,[2] Sarah Rees, MSc,[1] Mohamed Lockhat, PhD,[2] Lisa Spary, PhD,[1] Alan Watkins, PhD,[1] Vincent Gnanapragasam, PhD,[3] Simon J. Crabb, PhD[4]

[1]SAIL Databank, Swansea University Medical School, Swansea, UK; [2]Janssen-Cilag Ltd, High Wycombe, UK; [3]Divison of Urology, Department of Surgery & Cambridge Urology Translational Research and Clinical Trials Office, University of Cambridge, Cambridge, UK; [4]Cancer Sciences Unit, University of Southampton, Southampton, UK

Correspondence to: Maria Cristina Penaloza-Ramos, Janssen-Cilag Ltd, 50–100 Holmers Farm Way, High Wycombe, Buckinghamshire, HP12 4EG

Email: mpenalo3@its.jnj.com

Phone: 01494567329

Funding: Janssen-Cilag Ltd

Key words: Castration-resistant prostate cancer, metastases, non-metastatic, real world evidence, survival.

SUPPLEMENTARY DATA

Table S1: Patient demographics and baseline characteristics for the stringent cohort (compared with the main cohort)

	**Main nmCRPC cohort (n=1,465)**	**Stringent nmCRPC cohort (n=470)**	**All patients with PC excluding main nmCRPC cohort (n=36,556)**	**All patients with PC excluding stringent nmCRPC cohort (n=37,551)**
Age at, median (IQR)				
PC diagnosis	75.6 (69.2–80.4)	74.7 (68.5–79.8)	72.7 (65.9–79.6)	72.8 (65.9–79.6)
Initiation of ADT	76.6 (70.9–81.3)	76.0 (70.0–80.8)	74.3 (68.1–79.2)	76.2 (70.4–81.1)
nmCRPC inclusion	79.5 (73.6–84.6)	78.7 (72.6–83.6)	N/A	N/A
Development of metastases	78.1 (72.3–84.3)	78.5 (72.7–85.0)	75.4 (68.8–81.5)	75.5 (68.9–81.6)
Death	83.2 (77.0–88.4)	82.4 (76.4–87.8)	82.0 (75.8–87.1)	82.0 (75.9–87.2)
Treatment, n (%)				
ADT	1,465 (100.0)	470 (100.0)	15,446 (42.3)	16,441 (43.8)
Orchidectomy	<5 (<0.1)[a]	<5 (<0.1)[a]	>215 (~0.6)	>215 (~0.6)
Prostatectomy	18 (1.2)	7 (1.5)	4,343 (11.9)	4,354 (11.6)
Radiotherapy	189 (12.9)	140 (29.8)	5,152 (14.1)	5,201 (13.9)
PSA doubling time				
≤10 months	952 (65.0)	323 (68.7)	N/A	N/A
>10 months	513 (35.0)	147 (31.3)	N/A	N/A

	Main nmCRPC cohort (n=1,465)	Stringent nmCRPC cohort (n=470)	All patients with PC excluding main nmCRPC cohort (n=36,556)	All patients with PC excluding stringent nmCRPC cohort (n=37,551)
Gleason score				
≤6	187 (12.8)	48 (10.2)	9,220 (25.2)	9,359 (24.9)
7	269 (18.4)	90 (19.1)	6,457 (17.7)	6,636 (17.7)
8–10	347 (23.7)	134 (28.5)	4,223 (11.6)	4,436 (11.8)
Unknown	662 (45.2)	198 (42.1)	16,656 (45.3)	17,120 (45.6)
TNM score				
Tany N0 M0	64 (4.4)	11 (2.3)	5,536 (15.1)	5,589 (14.9)
Tany N1–2 M0	N/A	N/A	269 (0.7)	269 (0.7)
Tany Nany M1	N/A	N/A	619 (1.7)	619 (1.6)
Other	N/A	N/A	84 (0.2)	84 (0.2)
Unknown	1,401 (95.6)	459 (97.7)	30,048 (82.2)	30,990 (82.5)
Time in months, median (IQR)				
From PC diagnosis to nmCRPC	37.4 (20.3–66.9)	36.9 (20.5–64.2)	N/A	N/A
Duration of follow-up from nmCRPC diagnosis	37.6 (19.5–61.3)	41.1 (22.0–66.9)	N/A	N/A

[a]These results are presented as '<5' to ensure patient anonymity. ADT: androgen deprivation therapy; IQR: interquartile range; N/A: not available; nmCRPC: non-metastatic castration-resistant prostate cancer; PC: prostate cancer; PSA: prostate-specific antigen; TNM: tumor, node, metastasis.

Figure S1: Patient flow diagram

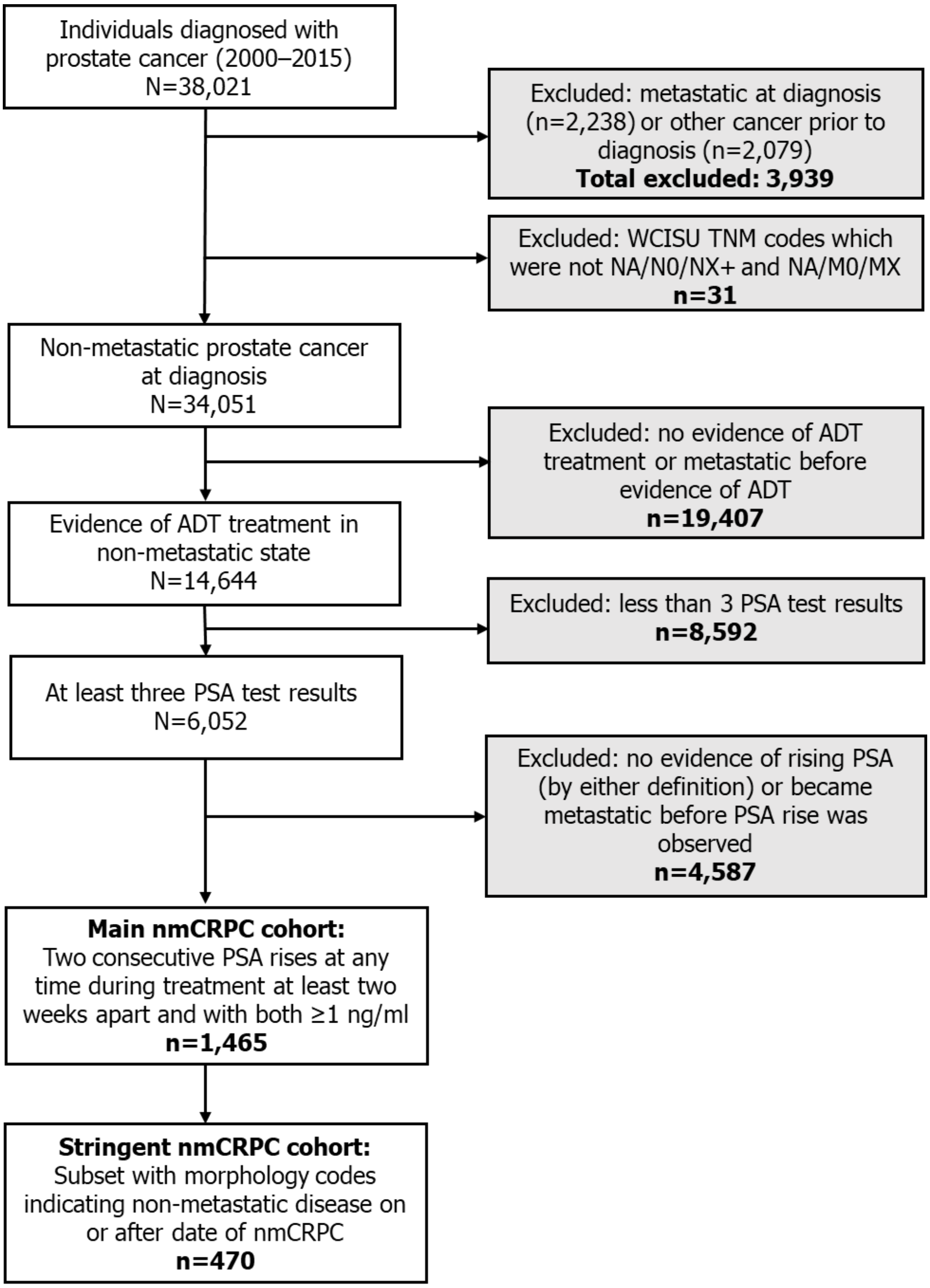

ADT: androgen deprivation therapy; nmCRPC: non-metastatic castration-resistant prostate cancer; PSA: prostate-specific antigen; TNM: tumor, node and metastasis code; WCISU: Welsh Cancer Incidence and Surveillance Unit.

DEFINITION OF VARIABLES

Date of primary malignant prostate cancer diagnosis

The data source was searched to identify all individuals where the first occurrence of a primary malignant prostate cancer (PC) diagnosis code (International Classification of Diseases [ICD]-10 code of C61% or Read code v2 of B46%) was between 01 January 2000 and 31 December 2015. The diagnosis date was the entry point to the overall primary malignant PC cohort (including individuals with cancers of all disease states). Welsh Cancer Incidence and Surveillance Unit (WCISU) was the primary data source as it contains a definitive diagnosis date. Where individuals were present in multiple datasets, the following priority sequence was used to determine date of diagnosis:

1. WCISU first diagnosis date
2. Wales Primary Care GPs (WLGP) first event date
3. Patient Episode Dataset for Wales (PEDW) first admission date – the reason for this is that PEDW records the dates on which individuals were admitted to hospital for treatment, but this is not necessarily the same as the date of diagnosis.

Diagnosis of other primary cancer

Cases were flagged to indicate patients diagnosed with another primary malignant cancer. The earliest and latest dates of primary malignant cancer diagnosis (other than PC) were included in the cohort dataset, to ensure that individuals undergoing treatment for other cancer could be excluded if required. All cancer codes from the primary malignant cancers ICD-10 chapter (for ICD-10 codes) and all primary cancer codes from the general practitioner (GP) Quality and Outcomes Framework (QOF) cancer indicator (for Read v2 codes) were flagged.

Diagnosis of secondary/metastatic cancer (including treatments indicating secondary/metastatic cancers)

Cases were flagged to show the earliest and latest dates of a diagnosis with secondary/metastatic cancer. All cancer codes from the secondary malignant cancers ICD-10 chapter (for ICD-10 codes) and all secondary cancer codes from the GP Quality and QOF cancer indicator (for Read v2 codes) were flagged. PEDW was

searched for ICD morphology codes (used in PEDW until December 2016). Morphology codes are identified in PEDW as any 6-character diagnosis code (any position) where the first two characters are 'M8' or 'M9'; metastatic morphology codes are defined as those where the sixth character is a '6'. The earliest and latest date of a morphology code indicating metastatic disease was recorded. Flags were created for individuals with codes for treatments indicating metastatic disease, with variables created for the earliest and latest treatment date.

Diagnosis of non-metastatic cancer

PEDW was searched for non-metastatic morphology codes (as above but where the sixth character is '0' [benign], '1' [uncertain whether benign or malignant], '2' [in situ] or '3' [malignant]). The earliest and latest date of a non-metastatic morphology code was recorded.

Androgen Deprivation Therapy

To qualify as 'on' Androgen Deprivation Therapy (ADT) for the purposes of selecting the non-metastatic castration resistance PC (nmCRPC) cohort, there should be a gap of no more than six months between medication prescription dates as recorded in the GP history. The start and end dates for the ADT continuous treatment period were defined as the date of first ADT prescription and the end date of the last prescription plus six months.

Non-metastatic disease

Metastatic disease was identified by the presence or absence of a number of codes. Any individuals with a Node (N) or Metastasis (M) code which indicated metastatic disease at time of diagnosis (according to the WCISU dataset) were excluded, as were any individuals with a WCISU morphology code indicating metastasis. In addition, the PEDW dataset was searched for morphology codes indicating metastatic or non-metastatic disease. Two levels of confidence were derived from these codes: the broader definition included anyone with no metastatic code in their history on or before the nmCRPC entry date (the main nmCRPC cohort); the more stringent definition included those individuals who had a definitive non-metastatic code on or after the nmCRPC date (the stringent nmCRPC cohort) in the absence of any codes indicating metastatic disease at this time.

Prostate specific antigen test results

The SPARTAN trial definition was adopted for a rising prostate specific antigen (PSA) concentration meeting the criteria for PSA progression; three PSA test results, with at least 1 week between each test, with increasingly high concentrations and with the latest PSA concentration ≥2 ng/ml [9]. The first date upon which PSA concentration rose (e.g. the second PSA test date) was adopted as the nmCRPC cohort entry date. All PSA test results had to be during ADT treatment periods.

Calculation of PSA doubling time

PSA doubling time (PSADT) was calculated using the same three PSA values used to determine nmCRPC diagnosis. The PSADT was calculated as the gradient of the linear regression of the log (base 2) of the three PSA values. No nadir correction was required. Arlen *et al.* suggested that a minimum interval of 4 weeks was required between PSA tests where nmCRPC diagnosis specified a 1 week interval [26]. Consequently, for consistency, the 1 week specification was maintained for the study cohort.

Survival to metastasis or death

Survival to metastasis or death was defined as the time from nmCRPC inclusion to the first detection of metastasis or death from any cause, whichever occurred first.

Follow-up time

Data for the PC cohort was extracted from WDS, to calculate follow-up time in SAIL and identify individuals lost to follow up.

DATASETS

Metadata are available in the references for each dataset.

WCISU is the national cancer registry of Wales and contains details of all individuals resident in Wales who have been diagnosed with cancer [27]. The WCISU dataset has an excellent level of coverage in terms of capturing individuals diagnosed with cancer, and clinical coding (ICD10) is 100% complete, but lacks detail about the ongoing patient journey, treatments over time or disease progression.

PEDW contains details about inpatient admissions using the ICD10 and Office of Population Censuses and Surveys Version 4 (OPCS4) standard coding systems [28]. It can be used to track a patient journey longitudinally as it contains a full history of contacts with inpatient care.

WLGP contains details of all contacts between individuals and their GP practice: observations, prescriptions, test results, symptoms, diagnoses etc. coded using Read codes (primarily v2) [29].

ADDE contains details of all deaths of individuals resident in Wales or resident elsewhere who died in Wales [30].

WDS provides the start and end dates of periods of registration with Welsh GP practices [16]. It is used to calculate follow-up periods, person-years at risk and to identify when individuals become lost to follow up.
